# Comparative Ability of *Oropsylla montana* and *Xenopsylla cheopis* Fleas to Transmit *Yersinia pestis* by Two Different Mechanisms

**DOI:** 10.1371/journal.pntd.0005276

**Published:** 2017-01-12

**Authors:** B. Joseph Hinnebusch, David M. Bland, Christopher F. Bosio, Clayton O. Jarrett

**Affiliations:** Laboratory of Zoonotic Pathogens, Rocky Mountain Laboratories, National Institute of Allergy and Infectious Diseases, National Institutes of Health, Hamilton, Montana, United States of America; Fondation Raoul Follereau, FRANCE

## Abstract

**Background:**

Transmission of *Yersinia pestis* by flea bite can occur by two mechanisms. After taking a blood meal from a bacteremic mammal, fleas have the potential to transmit the very next time they feed. This early-phase transmission resembles mechanical transmission in some respects, but the mechanism is unknown. Thereafter, transmission occurs after *Yersinia pestis* forms a biofilm in the proventricular valve in the flea foregut. The biofilm can impede and sometimes completely block the ingestion of blood, resulting in regurgitative transmission of bacteria into the bite site. In this study, we compared the relative efficiency of the two modes of transmission for *Xenopsylla cheopis*, a flea known to become completely blocked at a high rate, and *Oropsylla montana*, a flea that has been considered to rarely develop proventricular blockage.

**Methodology/Principal findings:**

Fleas that took an infectious blood meal containing *Y*. *pestis* were maintained and monitored for four weeks for infection and proventricular blockage. The number of *Y*. *pestis* transmitted by groups of fleas by the two modes of transmission was also determined. *O*. *montana* readily developed complete proventricular blockage, and large numbers of *Y*. *pestis* were transmitted by that mechanism both by it and by *X*. *cheopis*, a flea known to block at a high rate. In contrast, few bacteria were transmitted in the early phase by either species.

**Conclusions:**

A model system incorporating standardized experimental conditions and viability controls was developed to more reliably compare the infection, proventricular blockage and transmission dynamics of different flea vectors, and was used to resolve a long-standing uncertainty concerning the vector competence of *O*. *montana*. Both *X*. *cheopis* and *O*. *montana* are fully capable of transmitting *Y*. *pestis* by the proventricular biofilm-dependent mechanism.

## Author Summary

The ecology of plague is complex and its epidemiology is enigmatic. Many different flea species are able to transmit *Yersinia pestis*, the plague bacillus, and they can transmit in two different ways. Early-phase transmission can occur during the first week after a flea has fed on a diseased animal. Thereafter, transmission occurs only as bacterial growth in the flea foregut interferes with and eventually blocks blood feeding. Comparisons of the relative ability of different flea vectors to transmit have been problematic, and contradictory results have been reported for the ability of the ground squirrel flea *Oropsylla montana* to transmit beyond the early phase. Our results show that *O*. *montana* readily develops foregut blockage, and transmission by that mechanism was as good as or better than observed for *Xenopsylla cheopis*, a flea known to block at a high rate. In contrast, very few bacteria were transmitted in the early phase by either of these fleas compared to later times after infection, suggesting that early-phase transmission is pertinent only to highly susceptible animals. Improved characterization of the transmission patterns of different flea vectors will aid in modeling plague incidence in its various natural settings.

## Introduction

Once fleas had been implicated as important vectors of the plague bacillus, *Yersinia pestis*, attention quickly turned to the mechanism and dynamics of flea-borne transmission. The first experiments, conducted between 1904–1907 by members of the Indian Plague Research Commission and others, characterized what is now termed early-phase transmission [1–4]. When fleas that had just fed on a rodent with terminal plague bacteremia were collected and used to challenge naïve rodents, they remained infective for about a week, with the transmission rate peaking about three days after the infectious blood meal and then waning. Transmission very rarely occurred from challenge with a single flea, but challenges with 10 to 25 fleas resulted in a transmission rate of 20 to 70%. Because simultaneous challenge with multiple fleas was required, early-phase transmission has also been referred to as mass transmission. Although long assumed to be a form of mechanical transmission, recent studies suggest that it is not that simple [5]; however, the mechanism of this early-phase transmission has yet to be determined.

In 1914 a second mode of transmission was discovered [6, 7]. This later phase of transmissibility occurs after *Y*. *pestis* forms a dense biofilm in the proventriculus, a valve in the flea foregut [8]. As the biofilm grows and consolidates it interferes with the valvular function of the proventriculus. Because the infected valve is unable to close completely, blood flowing into the midgut can flow back out again, carrying bacteria along with it, and be regurgitated into the bite site. The proventriculus can eventually become completely blocked in some fleas, preventing any blood from reaching the midgut. Inflowing blood is stopped by the bacterial mass that fills the proventriculus, and transmission occurs when blood mixed with bacteria from the surface of the biofilm is refluxed back into the bite site. As they begin to starve, such completely blocked fleas make continuous, persistent attempts to feed, thereby increasing the probability of transmission. Each feeding attempt by a single blocked *X*. *cheopis* flea has a 25 to 50% transmission success rate [9–13].

The ecology of plague is complex, involving many different rodent-flea transmission cycles. The relative competence of several flea vectors has been examined to varying degrees (reviewed in [5]). In general, these studies indicate that different flea species vary in their potential to become infected and subsequently transmit *Y*. *pestis*. The comparative early-phase transmission efficiency of different flea vector species has been systematically evaluated in several recent studies [14–17]. However, comparisons of the ability to transmit after the early phase are problematic because a variety of experimental methods have been used. This has led to variable and sometimes contradictory results. For example, some studies indicate that the North American ground squirrel flea *Oropsylla montana* transmits very poorly by the proventricular biofilm-dependent mechanism [9, 10, 18, 19], whereas other studies show it to transmit as efficiently as *Xenopsylla cheopis*, the flea often cited as the most efficient transmitter by that mechanism [12, 20].

A second unresolved issue is the relative ecologic importance of the two transmission mechanisms. Transmission efficiency studies to date have concentrated on either the early-phase or the proventricular biofilm mechanism, making direct comparisons of their relative efficiency problematic, again due to the variety of experimental conditions used.

To begin to address these shortcomings, we developed a standardized, carefully controlled experimental model system to infect cohorts of fleas and to characterize infection, proventricular blockage, and transmission dynamics during a four-week period following a single infectious blood meal. Comparative results for *O*. *montana* and *X*. *cheopis* establish that both develop proventricular blockage and transmit efficiently via the biofilm-dependent mechanism. For both species, transmission efficiency in the early phase was lower than at later times after infection.

## Methods

### Flea infections

*Y*. *pestis* KIM6+ was inoculated from frozen stocks maintained at the Rocky Mountain Laboratories (RML) into 5 ml of brain-heart infusion (BHI) broth supplemented with 10 μg/ml hemin and incubated at 28°C. After overnight incubation, 1 ml of this culture was used to inoculate 100 ml of BHI that was incubated at 37°C for 18 to 19 hours without aeration. The KIM6+ strain lacks the *Yersinia* virulence plasmid and is attenuated in mammalian virulence but infects fleas normally [21]. *O*. *montana* were from a laboratory colony originally established at the CDC, Fort Collins [22]and maintained at RML since 2011. *X*. *cheopis* colonies were derived from fleas collected in Los Angeles, CA or Baltimore, MD and have been maintained at RML for ~ 10 to 25 years, respectively. Fleas were pulled randomly from colonies and starved for four days. Groups of about 300 fleas were allowed to feed through a mouse skin affixed to an artificial feeding device [21] containing 5 ml of heparinized mouse blood (collected from RML Swiss Webster mice on site) containing ~1 x 10^9^/ml *Y*. *pestis* KIM6+, or with KIM6+ that had been transformed with pAcGFP1 (Clontech; Mountain View, CA), a plasmid containing a constitutively expressed green fluorescent protein (GFP) gene. After the 1-hour feeding period, fleas were collected and immobilized by placing the tube containing them on ice. Fleas were then arrayed on a chill table (BioQuip; Rancho Dominguez, CA) under a dissecting microscope, and only those that had taken an infectious blood meal, evidenced by the presence of fresh red blood in the midgut, were kept and used for experiments. A sample of 20 female fleas was placed at -80°C for later determination of the initial infectious dose.

### Flea blockage, mortality, and infection rates

In conjunction with experiments to assess proventricular blockage, a second group of fleas from the same cohort used for infection was allowed to feed the same day on sterile mouse blood to serve as uninfected controls. After feeding, ~100 infected and control fleas (equal numbers of males and females) were put into separate flea capsules [23] that were kept at 21°C and 75% relative humidity. These fleas were allowed to feed on neonatal mice for 1 hour on days 2, 6, 9, 13, 16, 20, 23, and 27 after infection. An additional sample of 20 infected female fleas was maintained identically but collected and placed at -80°C on day 7 after infection. A shallow layer of sterile sawdust was added to the bottom of capsules containing *O*. *montana*. Following each maintenance feed, infected fleas were collected and examined microscopically for evidence of proventricular blockage, diagnosed by the presence of fresh red blood in the esophagus only, with none in the midgut. Blocked fleas were segregated into a separate capsule after diagnosis for separate maintenance. Mortality was also recorded on each maintenance feed day. A sample of 20 surviving female fleas was placed at -80°C when the experiment was terminated on day 28. Three independent blockage experiments were conducted for both *O*. *montana* and *X*. *cheopis*. After being diagnosed as blocked, *O*. *montana* fleas infected with the GFP+ strain were dissected and examined by fluorescence microscopy to verify proventricular blockage.

### Determining *Y*. *pestis* CFU in infectious blood meal and infected flea samples

Dilutions of the infectious blood meal were plated on blood agar to determine the *Y*. *pestis* CFU/ml. Samples of fleas that had been collected at different times after infection and stored at -80°C were thawed, surface sterilized, individually triturated, and then dilutions were plated in BHI soft agar overlays [24]. All plates were incubated at 28° C for 48 hours prior to colony counts.

### Monitoring transmission dynamics

For transmission experiments, fleas that took an infectious blood meal were housed as described above. On days 3, 10, 17, 24, and 31, ~200 fleas (approximately equal numbers of males and females) were allowed to feed on sterile defibrinated rat blood (BioreclamationIVT, New York) in the artificial feeding system. After 90 min, fleas were collected and examined microscopically as described above to determine how many had fed, and of those, how many were completely or partially blocked. A sample of 20 unblocked female fleas that had fed were placed at -80°C. Day 3 was the first feeding opportunity after the infectious blood meal and represents early-phase transmission. In addition to the transmission test artificial feedings, fleas were also fed on neonatal mice on days 6, 13, 20, and 27.

Immediately after each transmission test feeding, blood was removed and the interior of the feeder was washed ten times with 3 ml PBS. The entire volume of blood was distributively plated on blood agar plates. Pooled washes were centrifuged 9,800 ×*g* for 30 min, most of the supernatant removed and the remainder was mixed thoroughly to resuspend any bacteria and spread onto blood agar plates. During the period of peak transmission by *O*. *montana* (10 to 24 days after infection) dilutions of the blood and wash samples, instead of the entire sample, were plated. The external (haired) surface of the mouse skin membrane was disinfected with ethanol, cut into small pieces that were added to 1 ml PBS in a lysing matrix H tube and subjected to 2 min treatment in a FastPrep homogenizer (MP Biomedicals, Santa Ana, CA) to dislodge any transmitted bacteria associated with the interior surface of the mouse skin. Skin sample supernatants were pooled and centrifuged at 20,000 ×*g* for 15 min. Most of the supernatant was discarded and the remainder was vigorously mixed to resuspend any bacteria and then plated on blood agar. Three independent transmission experiments were conducted with both *O*. *montana* and *X*. *cheopis*.

### Flea proventriculus and esophagus measurements

Fleas were placed in PBS on a glass microscope slide and dissected with a set of fine forceps. The flea exoskeleton was removed and an 18 x 18 mm glass cover slip was gently placed over the top of the digestive tract. Images of flea digestive tracts were obtained using a Nikon Eclipse E800 microscope with an Olympus DP72 camera and cellSens imaging software. To calculate an esophagus:proventriculus (E/PV) width ratio, the width of the base of the esophagus was measured above the proventricular spines, where the musculature ends, and the proventriculus was measured at its widest position, near the midpoint of the valve.

### Statistical analysis

All analyses were performed using GraphPad Prism 6 (GraphPad Software Inc., La Jolla, Ca.). Statistical tests used and relevant *P* values are indicated in the figure legends.

### Ethics statement

All experiments involving animals were approved by the Rocky Mountain Laboratories, National Institute of Allergy and Infectious Diseases, National Institutes of Health Animal Care and Use Committee (protocols 13–036 and 14–006) and were conducted in accordance with all National Institutes of Health guidelines.

## Results

### Comparative infection and blockage rates of *O*. *montana* and *X*. *cheopis*

Groups of fleas were infected by allowing them to feed on highly bacteremic blood in an artificial feeding device, and thereafter provided twice-weekly maintenance feeds on uninfected mice for four weeks. Immediately after each maintenance feed, fleas were examined individually for the development of proventricular blockage, indicated by the presence of fresh red blood in the esophagus but not in the midgut.

**Fig 1:**
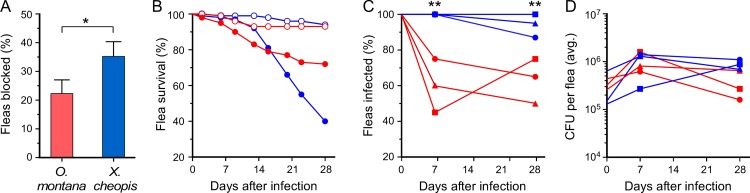
Comparative blockage and infection rates of *O*. *montana* and *X*. *cheopis* during a four-week period after a single infectious blood meal. (**A**) Percentage of fleas that developed complete proventricular blockage. The mean and SD are indicated. (**B**) Mortality rate of uninfected (open symbols) and infected (closed symbols) *O*. *montana* (red symbols) and *X*. *cheopis* (blue symbols). (**C**) Percentage of *O*. *montana* (red symbols) and *X*. *cheopis* (blue symbols) still infected 7 and 28 days after an infectious blood meal. (**D**) Mean bacterial load in infected *O*. *montana* (blue symbols) and *X*. *cheopis* (red symbols) immediately after the infectious blood meal (day 0) and at 7 and 28 days after infection. The cumulative results of three independent experiments (n = 99 to 113 fleas each) are shown in (**A, B**); the results of each of the three experiments are plotted in (**C, D**). *P = 0.0003; **P < 0.0001 by Fisher’s exact test (two-tailed).

**Fig 2:**
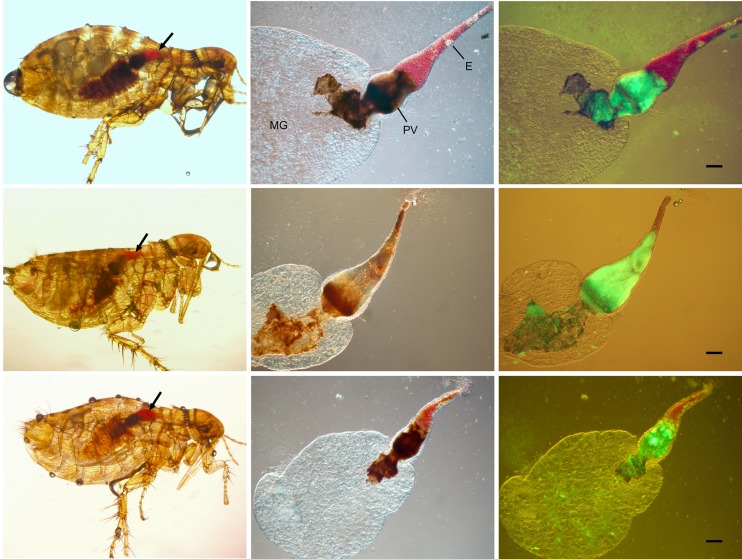
*Y*. *pestis* causes complete proventricular blockage in *O*. *montana* fleas. The left panels show the typical blockage phenotype in *O*. *montana* fleas immediately after a feeding attempt. Fresh red blood (arrows) that was unable to enter the midgut (MG) is seen in the esophagus (E). The fleas were infected with GFP-expressing *Y*. *pestis*. The middle and right panels are images of the dissected digestive tracts from the same fleas visualized by DIC and a combination of DIC + fluorescence microscopy, respectively, and confirm the presence of a dense *Y*. *pestis* biofilm that completely fills and blocks the proventriculus (PV). Scale bars = 100 μm.

**Fig 3:**
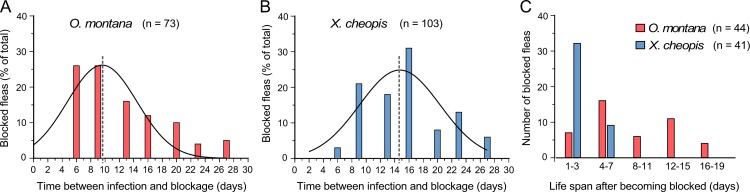
*O*. *montana* blocks earlier and survives longer after becoming blocked than *X*. *cheopis*. Histograms of the temporal incidence of complete blockage in *O*. *montana* and *X*. *cheopis* (**A**, **B**) and their life span after becoming blocked (**C**). Dashed lines indicate the mean; curve fitting used a Gaussian model of the frequency distribution data. Blocked *O*. *montana* survived significantly longer than blocked *X*. *cheopis*; *P* < 0.0001 by log-rank (Mantel-Cox) test.

Contrary to some previous reports, we found that *O*. *montana* readily became blocked, but at a lower incidence than *X*. *cheopis* (mean blockage rates 22% and 36%, respectively; Fig 1A and Fig 2). However, *O*. *montana* tended to become blocked sooner (mean = 9.6 days after infection) than *X*. *cheopis* (mean = 14.7 days) and to survive longer after becoming blocked (Fig 3). Blocked *O*. *montana* fleas survived up to 2 weeks after being diagnosed (range = 1 to 16 days; mean = 7 days; median = 4 days), whereas blocked *X*. *cheopis* survived a maximum of 4 days (range = 1 to 4 days; mean = 2 days; median = 1 day). The mortality rate of uninfected control fleas at 28 days was 6 to 7% for both species (Fig 1B). Since blocked fleas die from starvation, the excess mortality of infected fleas is a surrogate indicator of blockage. The majority of the mortality of infected *X*. *cheopis* (60%) and *O*. *montana* (28%) was in fact due to the death of blocked fleas, and reflects the difference in blockage rate between the two species.

The average number of *Y*. *pestis* that the fleas acquired in the infectious blood meal was 1.3 to 6.4 x 10^5^ for all experiments (Fig 1D). By 7 days after infection, a significantly greater percentage of *O*. *montana* than *X*. *cheopis* had eliminated the infection (Fig 1C). However, the average bacterial load in the infected fleas was equivalent for both species at all time points (Fig 1D).

### Transmission dynamics

In other experiments, groups of infected fleas were allowed to feed from a sterile blood reservoir at different times after infection. After feeding, fleas were collected and examined for evidence of feeding and proventricular blockage, and the number of *Y*. *pestis* that the fleas had transmitted was determined. The transmission test at 3 days after infection was the first feeding opportunity after the infectious blood meal, when transmission by the early-phase mechanism is maximum [1, 2, 5]. Between each weekly transmission test thereafter, fleas were provided a separate maintenance feed on an uninfected mouse. By the second transmission test on day 10 the fleas had had two uninfected blood meals since infection, and were beyond the early-phase transmission period. Thus, transmission on days 10 to 31 after infection was via the proventricular biofilm-dependent mechanism.

**Fig 4:**
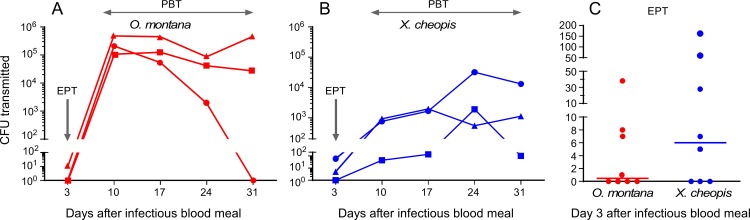
Transmission dynamics of *O*. *montana* and *X*. *cheopis* fleas during a four-week period after a single infectious blood meal. The number of *Y*. *pestis* CFU transmitted by *O*. *montana* (**A**) and *X*. *cheopis* (**B**) fleas by the early-phase (EPT, day 3) and proventricular biofilm-dependent (PBT) mechanisms in three independent experiments. See Tables 1 and 2 for details. (**C**) Number of *Y*. *pestis* CFU transmitted in the early phase (3 days after an infectious blood meal) by *O*. *montana* and *X*. *cheopis* in 8 independent experiments. Horizontal bars indicate the median. For these 16 EPT experiments, 103 to 202 fleas fed on day 3. The bacteremia level in the infectious blood meals ranged from 1.9 x 10^8^ to 3.0 x10^9^ CFU/ml.

Few *Y*. *pestis* were transmitted by the early-phase mechanism, and no transmission was detected in some experiments, even though over 100 fleas fed (Fig 4, Tables 1 and 2). Additional experiments were done to further evaluate early-phase transmission only (3 days after an infectious blood meal). The number of *Y*. *pestis* transmitted was below the detection level in 7 of 16 trials and ranged from 1 to 164 CFU in the nine others (Fig 4C). Fleas of both species transmitted many more bacteria after the early phase, peaking 10 to 24 days after infection at ~10^4^ CFU for *X*. *cheopis* and >10^5^ CFU for *O*. *montana*. Maxima of *Y*. *pestis* CFU transmitted (10 to 17 days after infection for *O*. *montana* and 17 to 24 days for *X*. *cheopis*) correlated with the mean time for proventricular blockage to develop (Fig 3A and 3B). At most time points after infection, the cumulative number of *Y*. *pestis* CFU transmitted by *O*. *montana* cohorts was 10-fold or more higher than the number transmitted by *X*. *cheopis* (Fig 4, Tables 1 and 2).

**Table 1:** *O*. *montana* transmission data summary.

Days P.I.^*a*^	No. fleas fed	% Infected	CFU/flea^*b*^	No. fleas blocked	No. fleas partially blocked	CFU transmitted^*c*^
	**Experiment 1** (1.9 x 10^8^ CFU/ml)^***d***^					
0	-	100	3.7 x 10^4^ ±2.9 x 10^4^	-	-	-
3	173	65	3.1 x 10^5^ ±5.3 x 10^5^	0	2	0
10	177	67	2.8 x 10^5^ ±2.9 x 10^5^	23	16	210,000
17	176	50	4.6 x 10^5^ ±4.1 x 10^5^	8	3	54,000
24	100	47	4.5 x 10^5^ ±3.3 x 10^5^	3	1	1,900
31	44	21	2.8 x 10^5^ ±1.1 x 10^5^	0	0	0
	**Experiment 2** (4.0 x 10^8^ CFU/ml)^***d***^					
0	-	100	2.4 x 10^5^ ±2.1 x 10^5^	-	-	-
3	136	35	2.1 x 10^5^ ±2.2x 10^5^	0	4	0
10	175	60	2.4 x 10^5^ ±4.9 x 10^5^	9	24	110,250
17	144	80	3.3 x 10^5^ ±5.9 x 10^5^	12	3	130,000
24	156	50	2.3 x 10^5^ ±2.9 x 10^5^	18	10	42,625
31	160	70	4.4 x 10^5^ ±3.0 x 10^5^	6	7	27,900
	**Experiment 3** (1.4 x 10^9^ CFU/ml)^***d***^					
0	-	100	2.9 x 10^5^ ±2.0 x 10^5^	-	-	-
3	202	40	1.8 x 10^5^ ±2.3 x 10^5^	0	3	7
10	188	65	6.0 x 10^5^ ±7.1 x 10^5^	20	20	418,900
17	195	90	5.0 x 10^5^ ±4.1 x 10^5^	14	17	392,700
24	136	80	4.6 x 10^5^ ±4.7 x 10^5^	3	9	76,800
31	177	80	5.3 x 10^5^ ±2.7 x 10^5^	7	5	403,600

^***a***^Days after the infectious blood meal. Day 0 indicates the day of infection; data for this day are included to show the initial infectious dose (no transmission test was done).

^***b***^Mean ± SD in infected fleas.

^***c***^Data used for Fig 4A.

^***d***^Conc. of *Y*. *pestis* in infectious blood meal on day 0.

**Table 2:** *X*. *cheopis* transmission data summary.

Days P.I. ^*a*^	No. fleas fed	% Infected	CFU/flea^*b*^	No. fleas blocked	No. fleas partially blocked	CFU transmitted^*c*^
	**Experiment 1** (1.1 x 10^9^ CFU/ml)^***d***^					
0	-	100	2.9 x 10^5^ ±1.9 x 10^5^	-	-	-
3	139	ND	ND	0	0	59
10	153	90	9.9x 10^5^ ±5.9 x 10^5^	2	1	760
17	136	ND	ND	9	7	1,660
24	128	ND	ND	11	11	32,000
31	98	85	5.7 x 10^5^ ±3.8 x 10^5^	8	12	13,200
	**Experiment 2** (2.0 x 10^9^ CFU/ml)^***d***^					
0	-	100	1.2 x 10^6^ ±4.0 x 10^5^	-	-	-
3	144	ND	ND	0	0	0
10	89	95	5.9 x 10^5^ ±3.4 x 10^5^	2	4	44
17	146	100	8.4 x 10^5^ ±9.7 x 10^5^	11	1	135
24	92	ND	ND	8	1	1,900
31	71	100	6.6 x 10^5^ ±5.1 x 10^5^	3	1	101
	**Experiment 3** (9.0 x 10^8^ CFU/ml)^***d***^					
0	-	100	4.0 x 10^5^ ±1.8 x 10^5^	-	-	-
3	156	95	1.0 x 10^6^ ±6.7 x 10^5^	0	0	5
10	138	90	1.4 x 10^6^ ±9.5 x 10^5^	2	19	921
17	142	100	7.1 x 10^5^ ±7.7 x 10^5^	9	11	1,950
24	145	100	8.7 x 10^5^ ±5.8 x 10^5^	14	6	550
31	126	100	8.0 x 10^5^ ±5.7 x 10^5^	8	2	1,120

^***a***^Days after the infectious blood meal. Day 0 indicates the day of infection; data for this day are included to show the initial infectious dose (no transmission test was done).

^***b***^Mean ± SD in infected fleas.

^***c***^Data used for Fig 4B.

^***d***^Conc. of *Y*. *pestis* in infectious blood meal on day 0.

ND = not determined.

### Comparative anatomy of the foregut of *O*. *montana* and *X*. *cheopis*

**Fig 5:**
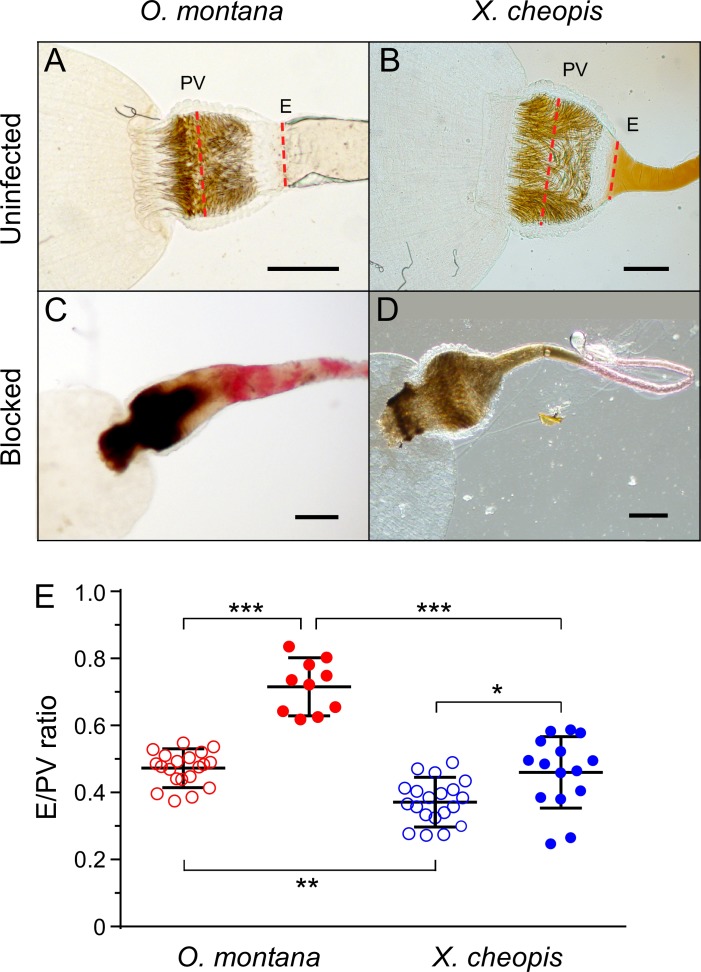
Proventricular blockage causes greater esophageal distension in *O*. *montana* than in *X*. *cheopis*. (**A-D**) Representative images of foreguts from dissected uninfected and blocked *O*. *montana* and *X*. *cheopis*. Dashed red lines indicate the location of esophageal (E) and proventricular (PV) measurements used to calculate the E:PV ratio of uninfected fleas (open symbols; n = 20) and blocked fleas (closed symbols; n = 10 to 15) in (**E**); the mean and SD are indicated. *P < 0.05; **P < 0.01, ***P < 0.0001 by one-way ANOVA and Tukey’s multiple comparison test. Scale bars = 100 μm.

While examining the digestive tracts of dissected fleas, we noticed that the base of the esophagus, where it joins the proventriculus, appeared to be wider in *O*. *montana* than in *X*. *cheopis*. This was especially evident in blocked *O*. *montana*, in which the base of the esophagus was often grossly distended (Figs 2 and 5). To evaluate this quantitatively, we measured the esophageal and proventricular widths in uninfected and blocked specimens of the two species and calculated a ratio. The esophagus:proventriculus (E/PV) width ratio was significantly greater for *O*. *montana* than *X*. *cheopis* (Fig 5E). In blocked fleas of both species, the *Y*. *pestis* proventricular biofilm extended into the esophagus. This expanded the relative width of the esophagus in blocked vs. unblocked *O*. *montana* to a greater extent than in blocked vs. unblocked *X*. *cheopis*. In some blocked *O*. *montana*, the esophagus was nearly as wide as the proventriculus (mean E/PV ratio = 0.71; range = 0.60 to 0.84).

## Discussion

In 1911, McCoy demonstrated that *O*. *montana* was able to transmit *Y*. *pestis* during the early phase, and more recent work has indicated that *O*. *montana* and *X*. *cheopis* are comparable in early-phase transmission efficiency [16, 25–30]. *X*. *cheopis* has also consistently been shown to become blocked and transmit after the early phase. However, conflicting results have been reported for the rate at which *O*. *montana* becomes blocked after infection and its corresponding ability to transmit by the proventricular biofilm-dependent mechanism. Wheeler and Douglas found that *O*. *montana* was an even better vector than *X*. *cheopis* during the four-week period after the early phase [12, 20], but other studies of *O*. *montana* reported little or no blockage and transmission during this time frame [9, 10, 18, 19]. Based on the negative data, the recent literature now states that *O*. *montana* rarely becomes blocked [26, 31]. A first objective of this study was to resolve the contradictory conclusions of the earlier studies by using standardized methods to directly compare the post-infection blockage rates of *O*. *montana* and *X*. *cheopis*.

We found that obvious, complete blockage of *O*. *montana* is not a rare but a regular occurrence (Fig 2). Differences among *O*. *montana* strains used for infection experiments has previously been suggested as a potential cause for conflicting results [10]. However, our *O*. *montana* colony originated from one used for a study that reported a 0% blockage rate [19]. A more likely reason for discordant results stems from the very high mortality rate associated with the studies that report negative results for *O*. *montana* blockage. Two studies that reported a 0 to 3% blockage rate also recorded a 23 to 59% die-off during the first week after the infectious blood meal, 58 to 79% at two weeks, and few fleas surviving after four weeks [10, 19]. In initial experiments, we also noted high mortality, even of uninfected control *O*. *montana*, and low and inconsistent blockage rates. Adult fleas maintained in proper conditions should live for a period of months, and we had previously observed that results with unhealthy *X*. *cheopis* cohorts (indicated by a 4-week mortality rate of >25% for uninfected control fleas) were unreliable and grossly underestimated the normal blockage rate observed for healthy *X*. *cheopis* (mortality rate of uninfected controls <10%). We found that unless *O*. *montana* were kept on a layer of sawdust, they became entangled via the long, curved pretarsal claws at the end of their legs, which led to stress-related mortality. The addition of a sawdust substrate to capsules containing *O*. *montana* (which is not necessary for *X*. *cheopis*) eliminated this problem and reduced uninfected control flea mortality at four weeks from 60 to 75% to <10%. Infected *O*. *montana* mortality was 20% at two weeks and 28% at four weeks after infection, correlating with the timing and incidence of blockage-induced mortality (Fig 1). A high mortality rate during the first week after infection indicates that the fleas were unhealthy or stressed, complicating the interpretation of the results. For this reason, experiments designed to compare long-term infection and transmission dynamics should include uninfected control fleas to verify normal viability. Different flea species may require different maintenance conditions to maintain health. The consistent high blockage and transmission rates reported for *X*. *cheopis* compared to other species may in part be due to the fact that *X*. *cheopis* is more easily adaptable to the laboratory.

A second objective was to compare the vector efficiency of *O*. *montana* and *X*. *cheopis* by the proventricular biofilm-dependent mechanism. The vector efficiency of a given flea species is a product of several factors, including infection potential (the percentage of individuals that become infected after feeding on blood containing *Y*. *pestis*), vector potential (the % of infected fleas that develop a transmissible infection; *i*.*e*., the % of *infective* fleas), and the transmission potential (the average number of transmissions effected by a group of infective fleas) [12, 32]. We measured the infection potential directly and found that it was lower for *O*. *montana* (45 to 75%) than for *X*. *cheopis* (87 to 100%; Fig 1C). This is consistent with previous reports that *O*. *montana* clears itself of infection at a higher rate than *X*. *cheopis* [10, 19, 20]. Although complete blockage is not required for transmissibility, the blockage rate is often used as a surrogate marker for vector potential by the proventricular biofilm-dependent mechanism. Like the infection rate, the blockage rate was also lower for *O*. *montana* (17 to 25%) than for *X*. *cheopis* (30 to 40%); however, blockage rates of stably infected fleas (rather than the total that took an infectious blood meal) were roughly equivalent for the two species. In this study, we monitored transmission by a population of infected fleas, which allowed an overall comparison of transmission dynamics and kinetics. To directly calculate the vector efficiency, it will also be necessary to monitor the vector potential and transmission potential of individual fleas after infection. We are currently adapting our model system for this purpose.

Despite its lower infection and blockage rates, *O*. *montana* transmitted greater numbers of *Y*. *pestis* than did *X*. *cheopis* in mass transmission experiments, and transmission peaked earlier. Several factors could account for these results. *O*. *montana* developed proventricular blockage sooner than *X*. *cheopis*, and blocked *O*. *montana* survived significantly longer than blocked *X*. *cheopis*. The blocking-surviving potential, defined as the mean day of death after becoming blocked divided by the mean day of becoming blocked after an infectious blood meal, has been described as an important component of flea vector efficiency [11, 18]. Based on the results shown in Fig 3, the calculated blocking-survival potential of *O*. *montana* (0.73) is 5-fold higher than that of *X*. *cheopis* (0.14). It is also important to note that, as mentioned previously, complete blockage is not required for transmission—partially blocked fleas can also transmit efficiently [7]. Partially blocked fleas, with fresh blood in the esophagus but some also in the midgut, were observed frequently during transmission trials (Tables 1 and 2). Complete blockage appeared to develop more gradually in *O*. *montana* or was more ephemeral, as sometimes a small amount of blood appeared to seep through into the midgut when a flea previously diagnosed as completely blocked fed again.

Differences in foregut anatomy may also enhance transmission efficiency of *O*. *montana*. The esophageal-proventricular junction is much broader in blocked *O*. *montana* than in blocked *X*. *cheopis* (Fig 5). The increased esophageal distension observed in fully blocked *O*. *montana* would be expected to expose a greater surface area of the infectious biofilm to contact with incoming blood during a feeding attempt, potentially enhancing regurgitative transmission. This may in part account for the greater number of CFUs recovered from *O*. *montana* mass transmission experiments. We have hypothesized that foregut anatomical differences may also account for the larger numbers of *Y*. *pestis* transmitted by *X*. *cheopis* than by the cat flea *Ctenocephalides felis* [24].

A third objective of this study was to evaluate, in the same cohorts of infected fleas, the relative importance of the two transmission mechanisms. The transmission efficiency of both *O*. *montana* and *X*. *cheopis*, defined here as the number of *Y*. *pestis* transmitted per infective flea, was very low by the early-phase mechanism compared to later proventricular biofilm-dependent transmission. In half of the day-3 early-phase transmission trials, no *Y*. *pestis* CFUs were recovered from the blood reservoir fed upon by >100 fleas. Based on trials in which we added known numbers of *Y*. *pestis* to the blood in the feeding system, the expected recovery rate is ~95%. Thus, we estimate that 0 to 5 CFU were transmitted in the negative early-phase transmission experiments. Our results are in line with previous work indicating that early-phase transmission is inefficient. In 1907 the Indian Plague Commission reported that only 1 of 67 fleas that had fed on a septicemic plague rat and then individually placed on naïve rats transmitted plague in the early phase [1]. Recent studies estimate a ~0 to 10% probability of a single *O*. *montana* or *X*. *cheopis* flea transmitting by the early-phase mechanism [16, 26–30]. These estimates were based on both disease incidence and seroconversion following challenge by groups of ten fleas, indicating that the number of *Y*. *pestis* transmitted was sometimes at or below the LD_50_, estimated at 1 to 10 CFU for the highly susceptible laboratory mice used. The bite of a single blocked *X*. *cheopis* results in transmission 25 to 50% of the time [9–12]. The number of CFU transmitted by the bite of a blocked *X*. *cheopis* is highly variable, ranging from <10 to several thousand [10, 13]. No data are available on the transmission efficiency of partially blocked fleas or the number of CFUs they transmit. Our system allowed us to monitor transmission by cohorts of infected fleas (“mass” transmission). This allowed an overall comparison of transmission dynamics and kinetics and vector potential at the population level, but not the transmission rate (the percentage of fleas that transmitted) or transmission potential (defined above). Consistent with our results, however, a recent study estimated that the percent transmission efficiency of an individual *O*. *montana* flea is lower in the early phase compared to later time points after infection [28].

Early-phase transmission has been proposed to largely account for epizootic spread by flea vectors that purportedly do not readily become blocked [33, 34]. However, it is recognized that comparative data regarding transmission by the proventricular biofilm (“blockage”) mechanism are limited and problematic, and warrant reexamination [5, 20, 35]. A variety of experimental conditions and designs have been used with respect to infectious blood meal source, infectious dose, and flea maintenance conditions, all of which are known to influence infection and transmission dynamics. In some cases, small numbers of fleas were used that were likely poorly adapted to laboratory conditions. As in the case of *O*. *montana*, results have sometimes been inconsistent. Furthermore, early-phase transmission has only been demonstrated from fleas that fed on blood with a very high bacteremia to highly susceptible laboratory rodents (ID_50_ <10 CFU) [36]. The California ground squirrel, the major host of *O*. *montana*, reportedly has an ID_50_ of >250 CFU [37]. Based on the previous reports that *O*. *montana* rarely blocks or transmits beyond the early phase, a recent study that modeled *O*. *montana*-ground squirrel plague made the assumption that only early-phase transmission was important, and that transmission beyond that was negligible [38]. Our results indicating that very few bacteria are transmitted early (less than the reported ID_50_ of ground squirrels), but that subsequent transmission is robust, suggest that the converse assumption is probably more realistic. However, factors specific to different ecological settings and host and vector populations may also affect transmission dynamics.

In this study, we present a standardized, stringently controlled model system to more reliably compare vector efficiency and to monitor transmission dynamics of a population of infected fleas. Key elements of this experimental system and their rationale include: 1) Fleas are infected on a specific blood source containing comparable concentrations of *Y*. *pestis*. Both the type of blood used and the bacteremia level significantly affect the infection potential and incidence of blockage [13, 19, 39]. Use of an artificial feeding device allows the infectious blood meal to be matched in different experiments. 2) A subset of the same flea cohort used for infection is fed on sterile blood of the same type for use as uninfected controls. Infected and uninfected fleas are kept in the same environment and provided the same type and frequency of maintenance feeds. The mortality of uninfected control fleas after 4 weeks should be low. If not, the fleas were physiologically stressed and results based on them are unreliable. Mortality of infected fleas should also be recorded, as it is a surrogate indicator of blockage-induced starvation. 3) Fleas are individually examined immediately after the infectious blood meal and only those that took a full blood meal are included in the study. The mean initial infectious dose acquired by the fleas is determined from a sample of these fleas, collected immediately after the infectious blood meal. Some flea species may be reluctant to feed on other than their natural blood source, resulting in a lower infectious dose and subsequent lower infectivity rate. 4) Fleas are examined microscopically immediately after each maintenance feed for evidence of partial or complete proventricular blockage using good quality optics and light source. Fluorescence microscopy of the digestive tract dissected from fleas infected with GFP-expressing *Y*. *pestis* is very useful to determine proventricular blockage status. This is particularly helpful for fleas that have a darkly pigmented exoskeleton that is less transparent to direct microscopic visualization of the esophagus, proventriculus, and midgut [24]. 5) Infection rate and bacterial load are monitored by plate counts of flea samples collected at different times after the infectious blood meal. 6) Transmission dynamics are monitored for a population that received the same infectious blood meal during a timeframe that encompasses both modes of transmission.

Our results resolve a long-standing controversy about the susceptibility of *O*. *montana* to become blocked and to transmit *Y*. *pestis* by the proventricular biofilm-dependent mechanism. We previously used this experimental system to show that *C*. *felis*, normally a poor vector by either mechanism, readily becomes blocked and transmits if its usual daily feeding behavior is altered [24]. The transmission dynamics of other flea vector species can be systematically reevaluated by using this system, with the important prerequisite that appropriate laboratory maintenance conditions can be established for them.
