# Correction: Comparative Ability of *Oropsylla montana* and *Xenopsylla cheopis* Fleas to Transmit *Yersinia pestis* by Two Different Mechanisms

**DOI:** 10.1371/journal.pntd.0008344

**Published:** 2020-05-28

**Authors:** B. Joseph Hinnebusch, David M. Bland, Christopher F. Bosio, Clayton O. Jarrett

In this article [1], the legend for Fig 1D incorrectly notes which species is represented by each color symbol in this figure panel. See below for the correct Fig 1 legend.

**Fig 1:**
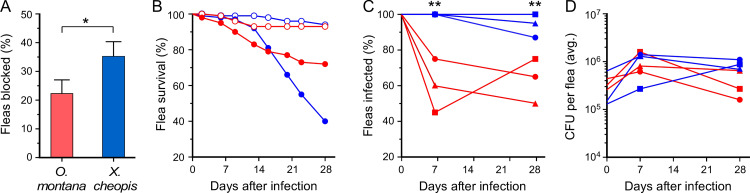
Comparative blockage and infection rates of O. montana and X. cheopis during a four-week period after a single infectious blood meal. (A) Percentage of fleas that developed complete proventricular blockage. The mean and SD are indicated. (B) Mortality rate of uninfected (open symbols) and infected (closed symbols) *O*. *montana* (red symbols) and *X*. *cheopis* (blue symbols). (C) Percentage of *O*. *montana* (red symbols) and *X*. *cheopis* (blue symbols) still infected 7 and 28 days after an infectious blood meal. (D) Mean bacterial load in infected *O*. *montana* (red symbols) and *X*. *cheopis* (blue symbols) immediately after the infectious blood meal (day 0) and at 7 and 28 days after infection. The cumulative results of three independent experiments (n = 99 to 113 fleas each) are shown in (A, B); the results of each of the three experiments are plotted in (C, D). *P = 0.0003; **P < 0.0001 by Fisher’s exact test (two-tailed).

In addition, the Data Availability Statement for this article is updated to, “The underlying data supporting results reported in this article are available upon request.”

The authors apologize for the errors in the published article.
